# Dietary Patterns and Birth Weight—a Review

**DOI:** 10.3934/publichealth.2014.4.211

**Published:** 2014-11-03

**Authors:** Marte K.R. Kjøllesdal, Gerd Holmboe-Ottesen

**Affiliations:** Institute of Health and Society, University of Oslo, Oslo, Norway

**Keywords:** low birth weight, small for gestational age, dietary patterns, dietary habits, pregnancy, food frequency questionnaire, fetal life conditioning

## Abstract

Being born with low birth weight (LBW) is recognized as a disadvantage due to risk of early growth retardation, fast catch up growth, infectious disease, developmental delay, and death during infancy and childhood, as well as development of obesity and non-communicable diseases (NCDs) later in life. LBW is an indicator of fetal response to a limiting intrauterine environment, which may imply developmental changes in organs and tissue. Numerous studies have explored the effect of maternal intake of various nutrients and specific food items on birth weight (BW). Taking into account that people have diets consisting of many different food items, extraction of dietary patterns has emerged as a common way to describe diets and explore the effects on health outcomes. The present article aims to review studies investigating the associations between dietary patterns derived from *a posteriori* analysis and BW, or being small for gestational age (SGA). A PubMed search was conducted with the Mesh terms “pregnancy” OR “fetal growth retardation” OR “fetal development” OR “infant, small for gestational age” OR “birth weight” OR “infant, birth weight, low” AND “diet” OR “food habits”. Final number of articles included was seven, all which assessed diet by use of food frequency questionnaire (FFQ). Five studies explored dietary patterns using principal component analyses (PCA), while one study used cluster analyses and one study logistic regression. The studies reported between one and seven dietary patterns. Those patterns positively associated with BW were labeled “nutrient dense”, “protein rich”, “health conscious”, and “Mediterranean”. Those negatively associated with BW were labeled “Western”, “processed”, “vegetarian”, “transitional”, and “wheat products”. The dietary patterns “Western” and “wheat products” were also associated with higher risk of SGA babies, whereas a “traditional” pattern in New Zealand was inversely associated with having a SGA baby. The dietary patterns associated with higher BW or lower risk of having babies born SGA were named differently, but had similar characteristics across studies, most importantly high intakes of fruits, vegetables and dairy foods. Dietary patterns associated with lower BW or higher risk for giving birth to a SGA baby were characterized by high intakes of processed and high fat meat products, sugar, confectionaries, sweets, soft drinks, and unspecified or refined grains. All studies in this review were performed in high-income countries. More research is warranted to explore such associations in low and middle income countries, where underweight babies are a major health challenge many places. Furthermore, results from studies on associations between diet and BW need to be translated into practical advice for pregnant women, especially women at high risk of giving birth to babies with LBW.

## 1. Introduction

Low birth weight (LBW) is usually defined as birth weight of a live born infant below 2500 grams [1]. LBW infants define a heterogeneous group: some born too early, some born at term but being small for gestational age (SGA), and some both born too early and being SGA [2]. Birth weight (BW) has been associated with mortality and various health outcomes later in life. Being born with LBW is generally recognized as a disadvantage for the infant, increasing the risk of early growth retardation, fast catch up growth, infectious disease, developmental delay, and death during infancy and childhood [2]. It has also repeatedly been associated with obesity [3] and non-communicable diseases (NCDs) later in life, such as coronary heart disease [4]–[6] and diabetes [7], as well as with risk factors associated with these diseases, such as hypertension [8], glucose intolerance [6], and hyperlipidemia [9]. LBW can be seen as an indicator of fetal response to a limiting intrauterine environment, which leads to metabolic changes in the functioning of organs and tissue, not evident at birth. This response is proposed to be due to early programming, occurring in a critical period of fetal life and resulting in permanent changes in the structure or functioning of the body's organs and tissues [10].

Fetal conditions depend on an equilibrated interplay between maternal nutrition, placental transport, and fetal growth factors. Maternal malnutrition not only affects fetal growth directly through insufficient nutrient supply and changes in placental functions, but also through epigenetic changes in the fetal genome [11]. In the end of World War II, the Netherlands experienced a severe famine during the winter months. It has later been shown that babies being exposed to this catastrophe during fetal life, especially in the last trimester, were born with lower BW than babies born before or conceived after the famine [12]. Many studies have followed which explore the effect of maternal intake of various nutrients and specific food items on BW. A number of studies have found positive relationships between BW and maternal intake of certain food items such as milk [13],[14], fruits [14],[15], and green leafy vegetables [14], but diverging associations with intake of fish [16],[17]. Associations with maternal intake of different macro- [18]–[21] and micro nutrients [18],[22],[23] have also been investigated.

However, individual diets are usually composed of variety of different food items which contain a multitude of nutrients and phytochemicals that function both synergistically and interactively [24]. Many of them are also highly correlated, which makes it difficult to separate their effects. Thus, the cumulative effect of nutrients may be easier to detect than for single nutrients alone. For this reason, many studies during the last years have attempted to extract dietary patterns in order to better represent a holistic account of the diet and analyze the patterns in association with various outcomes. There are two main types of methods for extracting dietary patterns, the *a priori* approach, being based on prior knowledge of e.g. a healthy diet, and the *a posteriori* approach, which entails a data-driven, explorative way of describing such patterns. The *a posteriori* approach is dependent on the population under study and decisions made by the researcher during the process of extracting patterns from the data. The reproducibility and the generalization of studies using this approach may therefore be questioned. One previous review has discussed methodological aspects of studies reporting on dietary patterns, both those analyzed *a priori* and *a posteriori*, during pregnancy [25]. The authors concluded with recommendations to use food frequency questionnaires (FFQ) validated for pregnant populations, to especially consider the use of supplements, and to select adequate timing for data collection. The study also reported on associations between these dietary patterns and health-related maternal and infant outcomes, such as pre-eclampsia, maternal weight gain and glucose status, preterm birth, birth weight, spina bifida, and cleft palate. The review included four studies using the *a posteriori* approach*,* but only one which reported on associations between dietary patterns and BW. Since it was published, a number of studies exploring *a posteriori* dietary patterns among pregnant women have been undertaken, of which several explore the association with BW. Considering the new knowledge generated and the importance given to BW in the emerging pandemic of NCDs, the present review further explores and expands on the knowledge regarding the association between empirically derived dietary patterns and BW. Articles reporting on the association between dietary patterns derived *a posteriori* and BW and/or being born SGA, such as are indicators of impaired fetal growth, published up to March 2014, are presented. Firstly, the aims were to review the literature on what characterize the dietary patterns derived *a posteriori* which are associated with BW, and secondly to discuss the comparability of these patterns during pregnancy extracted from the different studies.

## 2. Materials and Method

**Figure 1.**
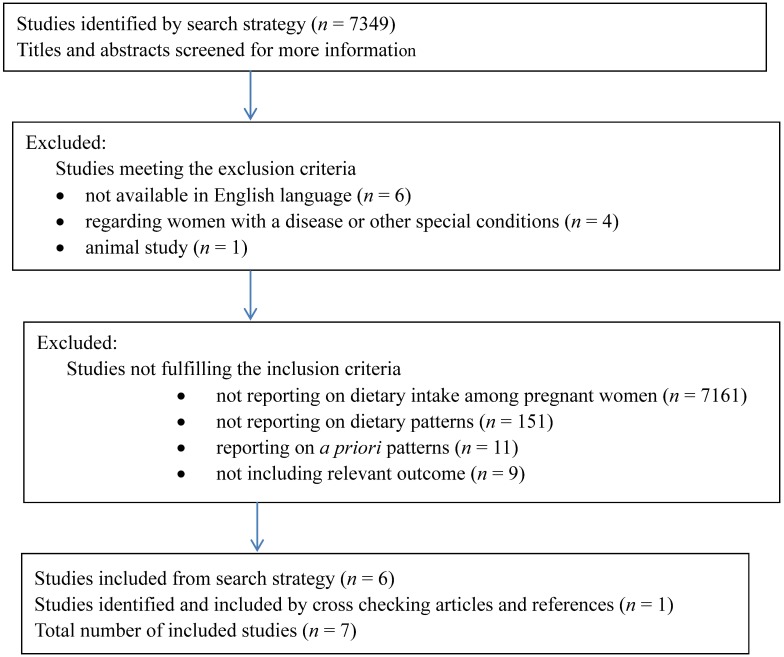
Flow chart describing the exclusion and inclusion of articles.

A PubMed search was conducted with the Mesh terms “pregnancy” OR “fetal growth retardation” OR “fetal development” OR “infant, small for gestational age” OR “birth weight” OR “infant, birth weight, low” AND “diet” OR “food habits” in articles published the last 20 years (from 1994 to March 2014) with available abstracts. The search resulted in 7349 articles. Inclusion criteria included articles reporting on the association between maternal dietary habits captured by dietary patterns derived *a posteriori* and/or BW or being born SGA. Exclusion criteria included articles not available in English language, reporting on pregnant women with special conditions, e.g. a certain disease, or on animals. All titles were noted, and abstracts or full papers were read for articles possibly fitting the review. A flow chart (Figure 1) describes the process. Final number of articles included was seven.

## 3. Results

### 3.1. Characteristics of the studies

Seven articles reporting on studies of the relationship between BW and/or SGA and dietary patterns explored with an *a posteriori* approach were included in the present review. The main characteristics of the studies are described in Table 1. Two articles were from the same study [26],[27], but each article employed different analyses to extract dietary patterns(PCA and logistic regression). One article was from 1994 [28], while the others were from 2008 and later. The studies presented here were conducted in the USA (Mexican Americans) [28], Denmark [29], UK [30], Netherlands [26],[27], New Zealand [31], and Japan [32]; thus, only reflecting dietary habits in high income countries. All studies assessed dietary data by FFQ. Most reported some type of validation of the questionnaire; either validation in a population other than pregnant women [26],[27],[32] or results were reported and correspond well to previous studies [28],[30]. Only the study from Denmark [29] reported to use a FFQ validated among the participants themselves.

**Table 1.** Description of the studies included in the present review.

Author	Country	Year	Study	Dietary assessment	N		Analysis for pattern extraction	Dietary patterns	Association with BW/SGA	Analysis adjustments
Wollf et al [27]	US (Mexican Americans)	1994	Hispanic HANES	FFQ, afterbirth	549		PCA	Nutrient dense	Pos. ass with BW	BMI
								Traditional	NS	Hemoglobin
								Transitional	Neg. ass. with BW	Gestational age at delivery
								Nutrientdiluted	NS	Maternal age
								Protein rich	Pos. ass. with BW	Infant gender
								High fat diary	NS	Smoking
								Mixed dishes	NS	
Knudsen et al [28]	Denmark	2008	Danish National Birth Cohort	FFQ, week 25	44612		PCA	Health conscious	Lower rate of SGA than “Western”	Maternal age
										Smoking
								Intermediate	Highest BW,	Mother`s height
									Lower rate of SGA than “Western”	Father`s height
								Western diet	Highest rate of SGA, lowest BW	
Northstone et al*[29]	UK	2008	Avon Longitudinal Study of Parents and Children	FFQ, week 32	14541	PCA		Health conscious	Pos. ass. with BW	unadjusted
								Traditional	NS	
								Processed	Neg. ass. with BW	
								Confectionery	NS	
								Vegetarian	Neg. ass. with BW	
Thompson et al [30]	New Zealand	2010	Auckland Birthweight Collaborative Study	FFQ, after delivery about first and last month of pregnancy**	1714	PCA		Junk	NS	Gestation, infant sex, smoking in pregnancy, maternal pre-pregnant height and weight, parity, ethnicity, maternal hypertension.
								Traditional	Neg. ass.withSGA	
								Fusion	NS	
Okubo et al [31]	Japan	2012	Osaka Maternal and Health Study	Diet History Questionnaire (week 5-39)	803	Cluster analysis		Meat and eggs		Maternal age, height, pre-pregnancy BMI, parity, gestational weight gain, week of gestation at survey, smoking, diet change last month, family structure, occupation, season, medical problems in pregnancy, baby`s sex
								Wheat products	Higher OR for SGA and lower BW than “rice, fish,& vegetables”	
								Rice, fish & vegetables		
Bouwland-Both et al [25]	Netherland	2012	Generation R Study	FFQ, month 3	847	PCA		Mediterranean	not reported***	Duration of last menstrual cycle, maternal age, maternal and paternal BMI and height, fetal gender, parity, educational level, smoking, folic acid supplement use, and blood pressure, SD score of crown-rump length
								Energy-rich	NS	
								Western	not reported***	
Timmermans et al [26]	Netherland	2012	Generation R Study	FFQ, month 3	3207	Regression residual method, to identify dietary patterns predicting intrauterine growth restriction		*Adherence to Mediterranean* diet:		Maternal age, parity, education, height, weight, smoking, folic acid use, vomiting, comorbidity, sex, gestational age
								High	Higher BW than low and intermediate	
								Intermediate		
								Low		

The questionnaires were distributed at different stages of pregnancy. In the Netherlands, mothers were asked to fill out a FFQ at the end of the first trimester, and to report the last three months [26],[27]. In Denmark, mothers filled in the FFQ at week 25, reporting from the last four weeks [29], and in the UK, dietary data were collected at week 32 [30]. In Japan, the diet history questionnaire was filled out at inclusion, which varied from gestational week 5 to 39 [32]. Mothers in New Zealand filled in the FFQ a short time after delivery, reporting from the first and the last month of pregnancy [31]. Finally, in the USA, mothers also filled in the FFQ retrospectively, but up to several years after delivery [28].

The number of food items and food groups covered by the FFQs varied from 57 in the American study [28] to 360 in the Danish [29]. In all but one study [31], the food items were collapsed into fewer groups before entered into analyses to extract dietary patterns. The unit for food groups entered into analyses was grams [26],[27],[29],[32] or frequency per week [28],[30]. One study [31] did not report which unit was used. Two studies [30],[32] reported to have collected data on dietary supplements; however, these data were not used when extracting dietary patterns.

Five studies [26],[28]–[31] explored dietary patterns using PCA. One study used cluster analyses [32] and one used logistic regression [27].

Associations between dietary patterns and BW/SGA were adjusted for a number of variables in all but one study. Variables commonly adjusted for included that of infant gender [26]–[28],[31],[32] and gestational age [27],[28],[31], maternal age [26]–[29],[32], pre-pregnancy anthropometrical measurements [26],[27],[29],[31],[32] and smoking habits [26]–[29],[31],[32], and indicators of socioeconomic position (SEP) [26],[27],[32]

### 3.2. Dietary pattern

The studies reported between one and seven dietary patterns each. The food items with high and low loadings on each pattern are shown in Table 2.Wollf et al [28] reported seven dietary patterns among Hispanic pregnant women: “nutrient dense”, “traditional”, “transitional”, “nutrient diluted”, “protein rich”, “high fat dairy”, and “mixed dishes”. From Denmark [29], two dietary patterns were reported, and the participants were grouped according to quintiles of those patterns. Those who were in the upper two quintiles of the first pattern, and those in the two lowest quintiles of the second, were labeled “Western diet." These women had the highest intake of high-fat dairy, refined grains, processed and red meat, animal fat, potatoes, sweets, beer, coffee, and high-energy drinks. Those who were in the lower two quintiles of the first pattern and in the upper two of the second pattern, were labeled “health conscious”. These women had the highest intake of fruits, vegetables, fish, poultry, breakfast cereals, vegetable juice, and water. Mothers who did not fit into these two categories were labeled “intermediate”. The women in the intermediate group had the highest intake of low-fat dairy and fruit juice. Intake of other foods was between the two other groups. Northstone et al [30] reported five dietary patterns among pregnant women in the UK: “health conscious”. “traditional”, “processed”, “confectionery”, and “vegetarian”. In New Zealand [31], three dietary patterns were reported: “junk”, “traditional”, and “fusion”. Okubo et al [32] reported three patterns from Japan: “meat and eggs”, “wheat products”, and “rice, fish, and vegetables”. Bouwland-Both et al [26] reported three dietary patterns from the Netherlands derived by PCA: “Mediterranean”, “energy-rich”, and “Western”, whereas Timmermanns et al [27] reported a “Mediterranean” pattern derived by the logistic regression from the same study.

**Table 2.** Description of food- and drink items characterizing the dietary patterns found in each study included in the present review.

Country	Dietary patterns	Food- and drink items
US (Mexican Americans)	Nutrient dense	Vitamin A rich fruits and vegetables, other fruits, low fat dairy products, other vegetables
	Traditional	Tortillas, legumes, high fat meats, sugar
	Transitional	Fats and oils, bread and cereals, other vegetables, high fat meats, sugar
	Nutrient diluted	Salty snacks, non-dairy, sugar
	Protein rich	Dairy desserts, low fat meats, processed meats
	High fat diary	High fat dairy products, soup, negative factor loadings for low fat dairy products
	Mixed dishes	Mixed dishes, soup, processed meats
Denmark	Pattern 1	Animal fat, margarine, processed meat, red meat, refined grains, eggs, potatoes, snacks, sweets, high-fat dairy
	Pattern 2	Vegetables, tomatoes, green leafy vegetables, fruit, fish, water, vegetable fats, poultry
UK	Health conscious	Non-white bread, bran based cereals, oat based cereal, fish, cheese, pulses pasta, rice, salad, fresh fruit, fruit juice, negative factor loadings for white bread
	Traditional	Potatoes, green leafy vegetables, other green vegetables, carrots, other root vegetables, peas
	Processed	White bread, meat pies, sausages/burgers, fried foods, pizza, eggs, baked beans
	Confectionery	Crispbreads/crackers, puddings, cakes/buns, sweets, chocolate, chocolate bars, crisps
	Vegetarian	Nuts, herbal tea, negative loadings for poultry, red meat
New Zealand	Junk	Ice cream, sweet biscuits, scones, cakes, sweetened cereals, crisps, pies, lollies, chocolate bars, ice blocks, and milo (chocolate drink)
	Traditional	Apples/pears, citrus fruit, kiwifruit/feijoas, bananas, green vegetables, root vegetables, peas/maize, dairy food/yogurt, water
	Fusion	Fruits, fried rice/noodles, boiled rice/pasta, fish/shellfish, milk, and negative loadings for tea/coffee, sherry/wine, hard cheeses
Japan	Meat and eggs	Beef, pork, processed meat, chicken, eggs, butter, dairy products
	Wheat products	Bread, confectioneries, fruit and vegetable juice, soft drinks
	Rice, fish, vegetables	Rice, potatoes, nuts, pulses, fruits, green and yellow vegetables, white vegetables, mushrooms, seaweeds, Japanese and Chinese tea, fish, shellfish, sea products, miso soup, salt-containing seasoning
Netherland	Mediterranean	Vegetables, legumes, pasta/rice, dairy, fish/shellfish, vegetable oils, alcohol, non-sweetened nonalcoholic beverages, negative loadings for processed meat
	Energy-rich	Bread/breakfast cereals, margarine, nuts, snacks/sweets, non-sweetened nonalcoholic beverages, negative loadings for sweetened nonalcoholic beverages
	Western	Potatoes, pasta/rice, dairy, fresh meat, processed meat, margarine, alcohol, negative loadings for nuts fish/shellfish
Netherland	Mediterranean	Pasta, rice, vegetable oils, fish, vegetables, alcohol, low intakes of meat, potatoes, fatty sauces

### 3.3. Dietary patterns associated with birth weight

All but one [31] study reported on the association between dietary patterns and BW. Positive associations with BW were reported for both the “nutrient dense” and the “protein rich” patterns [28]. For the “intermediate” group: between the “health conscious” and the “Western” pattern [29], the “health conscious” pattern [30], and also high adherence to the “Mediterranean” pattern [27]. Negative associations between dietary patterns and BW were reported for the “transitional” pattern [28], the “Western diet” [29], a “processed” and a “vegetarian” pattern [30], and for “wheat products” [32].

Bouwland-Both et al [26], reported only on the association between BW and the “energy-rich” pattern (non-significant), as this was the only pattern out of three which was significantly and positively associated with crown-rump length (CRL) in first trimester.

### 3.4. Dietary patterns associated with being small for gestational age

Three of the studies [29],[31],[32] included analyses of relationships between dietary patterns and SGA. Knudsen et al [29] reported highest rates of SGA babies among mothers with a “Western diet”. Thompson et al [31] found a negative association between having a SGA baby and a “traditional” dietary pattern. Finally, Okubo et al [32] reported higher odds for having babies born SGA among mothers belonging to the “wheat products” cluster.

## 4. Discussion

The present study has reviewed seven articles reporting on associations between dietary patterns during pregnancy and BW and/or risk of giving birth to a SGA baby in high-income countries. With the exception of one [26], all studies reported significant associations between dietary patterns and BW and/or SGA. Dietary patterns being positively associated with BW were labeled “nutrient dense”, “protein rich”, “health conscious”, and “Mediterranean”. Those negatively associated with BW were labeled “Western”, “processed”, “vegetarian”, “transitional”, and “wheat products”. The dietary patterns “Western” and “wheat products” were also associated with higher risk of SGA babies, whereas a “traditional” pattern in New Zealand was inversely associated with having a SGA baby.

The dietary patterns associated with higher BW or lower risk of SGA babies were named differently, but had similar characteristics across the studies. The “nutrient dense” [27], the “health conscious” [30] and the “traditional” [31] pattern all included fruits. The “nutrient dense” [28], the “traditional” [31], and the “Mediterranean” [27] patterns included vegetables, while the “health conscious” [30] included salad. Only the “protein rich” [28] pattern did not include vegetables or fruits. However, this pattern together with the “nutrient dense” may explain more of the variation in BW, as all other pattern associated with higher BW included dairy and/or fish together with vegetables and/or fruit. Most patterns also included dairy products in various forms, such as low fat dairy products in the “nutrient dense” pattern [28], dairy desserts in the “protein rich” pattern [28], cheese in “health conscious” pattern [30], and dairy food and yogurt in “traditional pattern” [31]. Furthermore, the “health conscious” [30] pattern had high loadings on non-white bread, bran- and oat based cereals, and pasta and rice, whereas the “Mediterranean” pattern [27] included pasta and rice. Two patterns, the “health conscious” [30] and the “Mediterranean” [27], had high loadings on fish. The “protein rich” pattern [28] had high loadings on low fat meats and processed meats, whereas the “Mediterranean” pattern had negative loadings for meat. Many elements of the dietary patterns associated with higher BW or lower risk of giving birth to SGA babies, are thus in line with findings from studies on the relationship between BW and single food groups, such as fruit and vegetables [14],[15], milk and other dairy products [13],[14], and fish [17].

In contrast, dietary patterns associated with lower BW or higher risk of having a SGA baby were characterized by higher intakes of processed meat and high fat meats, which had high loadings in the “transitional” pattern [28], the “Western” pattern [29], and the “processed” pattern [30]. Fats and oils had high loading in the “transitional” pattern [28], as did animal fat in the “Western” pattern [29]. Sugar, confectionaries, sweets, and soft drinks had high loadings in the “transitional” [28], the “Western” [29], and the “wheat products” [32] patterns. Unspecified or refined grains also had high loadings in the “transitional” [28], the “processed” [30], the “wheat products” [32], and the “Western” patterns [29]. These dietary patterns probably have low micronutrient content and correspond well to previously reported positive associations between the micronutrient content in maternal diets and increased fetal growth [14],[23]. However, there are some exceptions to this general finding, since high loadings were reported for vegetables in the “transitional” pattern [28] and for fruit in the “wheat product” pattern [32].

The dietary patterns associated with higher BW across different countries and in different stages of pregnancy had both similarities and differences. Dietary patterns derived *a posteriori* do not reflect optimal eating patterns, but rather how people compose their diet. Thus, variations in patterns between populations are to be expected. The findings from the present review, that dietary patterns characterized by high loadings of vegetables, fruit, and dairy products are associated with higher BW, are in accordance with studies reporting on relationships between single food items and BW. It can thus be questioned what dietary pattern analyses add to the big picture. However, studies using *a posteriori* dietary patterns may in addition confirm that vegetables, fruit and dairy products are important for BW when the overall diet is taken into account. It also underscores that all food items with a high factor loading may not be equally important in relation to the outcome under study. Thus, reviewing studies from several populations may be needed in order to capture the most important health related aspects of the dietary patterns. The consistency between the findings from studies on food groups/items as part of dietary patterns extracted *a posteriori* and studies investigating single foods or food groups strengthen the implications to be drawn from the association between diet and BW.

All studies in the present review focused on low birth weight as an unfavorable outcome, which it truly is. However, high birth weight (> 4500 gram) has also been shown to be detrimental, both in terms of conditions related to birth [33], for later child obesity, and future risk of non-communicable diseases, such as breast cancer [34],[35] and other cancers [34],[36]. One of the main risk factors for high BW is maternal obesity. Common dietary advice to prevent and reduce the overweight condition is to reduce intake of energy, through reducing intake of fat and also sugar [37]. Thus, foods that could contribute to a high BW are the same as the ones being part of a dietary pattern associated with low BW, as described in the present review. Overall, it seems that a diet rich in micronutrients, and low in “empty calories” from high-fat and high-sugar foods, promotes healthy development of the fetus and favorable BW.

BW is a common proxy of fetal growth, but other anthropometric measures indicate slightly different aspects of growth. The Generation R study from the Netherlands [26],[27] included the CRL measure in the first trimester and estimated fetal weight in the second and third trimesters. The findings showed that CRL was significantly associated with an “energy-rich dietary pattern*”,* but no association was seen between this pattern and fetal weight later in pregnancy or at birth. However, birth length, head circumference, and abdominal circumference are commonly measured and could perhaps have given other results in regard to dietary effect. In addition, measures of body composition, such as fat distribution and lean body mass of the newborn infant, can vary at any birth weight as a result of dietary quality. Apart from diet, both over- and under-nutrition of the mother, and ethnicity, may determine both body size and body composition of the baby. Yajnick et al [38] found that poor Indian mothers produced babies that were “thin-fat”, meaning that they had low BW, were shorter, had lower head and abdominal circumference, and had relatively lower lean body mass, but higher adiposity as compared to UK babies. The authors suggested that the reason for this finding was maternal B12 deficiency in combination with relatively high folate concentration due to a mostly vegan diet with low intake of foods from animal sources [39].

This review has summarized findings on associations between BW and/or being SGA and maternal dietary patterns in several countries. These findings may have been influenced by variations in proportions of LBW/SGA infants, as well as by use of different cut-offs for SGA and LBW between countries. Average BW is likely to differ between countries, and also between studies, due to differences in years of data collection. Two studies reported on average BW: in the USA, 3373 grams [28] and in Denmark, 3680 grams among boys and 3547 grams among girls [29]. Definitions of SGA were varied from < 2.5^th^ percentile [29] to 10^th^ percentile of BW [31]. SD scores and Z-scores for BW were defined using different standards [26],[29]. Despite the possibility of introduced bias, as all studies were performed in high-income countries, variations in BW are likely to be modest and the impact of such bias small. Conceivably, the impact of nutritional supply to the fetus may be larger in countries with widespread under-nutrition. Thus, results from similar studies in low income countries may have different outcomes.

All but one study made adjustments to the analyses of the relationships between dietary patterns and BW. However, only three articles reported adjustment for SEP. The Dutch study [26],[27] included education as an indicator of SEP, whereas the study from Japan [32] included both education and occupation. Associations have been demonstrated both between SEP and dietary habits [40],[41] and between SEP and BW [42]. Thus, SEP could therefore possibly be a confounder of the associations between dietary patterns during pregnancy and BW. However, significant associations between dietary patterns and BW were found also in the studies adjusting for SEP [27],[32].

None of the studies included dietary supplements as part of the dietary patterns, but one study [32] adjusted for use of supplements when exploring the associations with BW/SGA. Use of dietary supplements during pregnancy has previously been shown to have significant effect on BW [43]. Thus, use of supplements may be a confounding factor not accounted for in the present studies.

Estimating intake from FFQs may be a challenge, as both under- or over-reporting commonly occur. Moreover, a questionnaire based on recall may not capture all that is eaten. The *a posteriori approach* of analyzing dietary data implies that many decisions have to be made at several stages in the process. Differences in patterns between various studies could therefore be due to technical choices during the analyses, in addition to differences in data per se. In the studies included in this review, the time frame for data collection varied from as early as five weeks into pregnancy [32] to several years after birth [28], thereby covering different periods of the pregnancy. Earlier research suggests that maternal nutrition at different stages of gestation is affecting the fetus and its growth and development differently [12]. Another problem is the errors introduced when having to recall dietary intake years back. Memory may even be influenced by later happenings, such as a sick child. Finally, when comparing studies from several countries, dietary habits will vary due to cultural differences. In the present review, most studies were from countries with dietary habits that may have many shared characteristics, but the Japanese diet may vary considerably from the other more Western countries. However, despite technical and cultural differences between studies, dietary patterns associated with BW had substantial similarities across countries. However, extrapolating the results or the methods to low-income countries, often with a low variety of foods, may be challenging.

## 5. Conclusion

Despite differences in dietary patterns among groups of pregnant women in various countries, some common features were found in relationships between dietary patterns and BW. Most dietary patterns associated with higher BW were characterized by high intakes of vegetables, fruit, and of dairy products. Dietary patterns associated with low BW were often characterized by high loadings of processed and high-fat meat, fats and oils, and sugar rich products. All studies included in this review were done in high-income countries. More research is warranted to explore these associations in low and middle income countries, where the problem with underweight babies is a major health challenge. Results from studies on associations between diet and birth weight need to be translated into practical advice for pregnant women, especially for women at high risk of giving birth to babies with low BW. Furthermore, improved means to help women make healthy dietary changes before and during pregnancy is necessary, both in high, middle, and low income countries. This can be an important contribution to the efforts to reduce the risk of obesity and cardiovascular disease in future generations. A standardization of how to collect dietary data during pregnancy, with respect to timing of data collection during pregnancy and choice of method for data extraction, would make comparisons between populations more convenient and relevant.
